# Time-discrete SIR model for COVID-19 in Fiji

**DOI:** 10.1017/S0950268822000590

**Published:** 2022-04-07

**Authors:** Rishal Amar Singh, Rajnesh Lal, Ramanuja Rao Kotti

**Affiliations:** School of Mathematical and Computing Sciences, Fiji National University, Lautoka, Fiji

**Keywords:** COVID-19 in Fiji, reproduction number, SIR model, time-varying parameters

## Abstract

Using the data provided by Fiji's ministry of health and medical services, we apply an implicit time-discrete SIR (susceptible people–infectious people–removed people) model that tracks the transmission and recovering rate at time, *t* to predict the trend of the coronavirus disease 2019 (COVID-19) pandemic in Fiji. The model implied time-varying transmission and recovery rates were calculated from 4 May 2021 to 9 October 2021. The estimator functions for these rates were determined, and a short-term (30 days) forecast was done. The model was validated with observed values of the active and recovered cases from 11 October 2021 to 9 December 2021. Statistical results reveal a good fit of profiles between model simulated and the reported COVID-19 data. The gradual decrease of the time-varying basic reproduction number with values below one towards the end of the study period suggest the government's success in controlling the epidemic. The mean reproduction number for the second wave of COVID-19 in Fiji was estimated as 2.7818. The results from this study can be used by researchers, the Fijian government, and the relevant health policy makers in making informed decisions should a third COVID-19 wave occur.

## Introduction

A cluster of pneumonia infections was reported in Wuhan, China, in December 2019. Some of the first cases had regular contact with Wuhan wet markets that predominately traded live seafood [1]. Research revealed that the illness was caused by a newly discovered Coronavirus, subsequently named coronavirus disease 2019 (COVID-19) [1, 2]. The virus expanded throughout China and the rest of the globe, prompting the World Health Organization to designate the outbreak as a public health emergency of worldwide concern on 30 January 2020, and later, a global pandemic on 11 March 2020 [3]. Pacific Island countries were not spared of the spread and wrath of the virus. The first case in the region was discovered in French Polynesia on 10 March 2021 [4]. Eleven countries (Commonwealth of the Northern Marianas, Fiji, French Polynesia, Guam, New Caledonia, Papua New Guinea, Republic of the Marshall Islands, Samoa, Solomon Islands, Vanuatu and Wallis and Futuna) in the Pacific Island Countries and Territories (PICTs) have since reported cases and deaths.

**Fig. 1.**
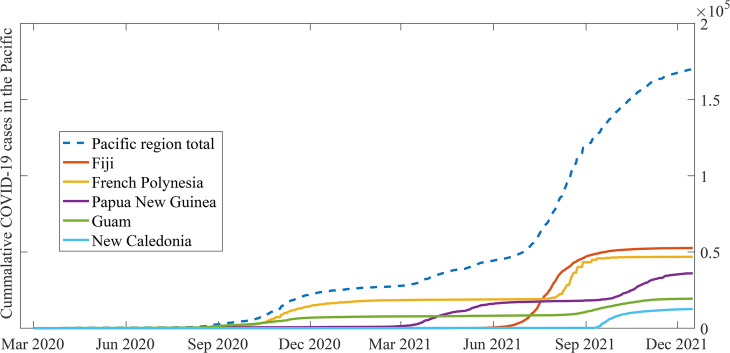
COVID-19 cases in the worst affected nations in the Pacific. The data of these PICTs is taken from [4].

As of 1 December 2021, the total number of cases in the PICTs stands at 167 695 with 2435 reported COVID deaths [4]. The worst affected nations were Fiji, French Polynesia, Papua New Guinea, Guam and New Caledonia. Figure 1 shows the cumulative cases from March 2020 to November 2021 of these PICTs. Countries such as American Samoa, Palau, Solomon Islands, Marshal Islands, Tonga, Vanuatu and Samoa were able to insulate themselves from the virus. Their geographical isolation from the rest of the world, combined with a peremptory response in the earliest periods of the pandemic by the respective governments through swift and an outright ban on inbound flights and ships helped achieve this. These total bans on border entry were gradually relaxed for citizens and cases were recorded from returning citizens who contracted the virus overseas. These citizens were put in strict isolation in quarantine facilities upon return. As of 1 December 2021, these seven PICTs had a combined COVID-19 population of 49, which constituted less than 0.03% of the total COVID population in the Pacific region [4].

Fiji, an archipelago of more than 330 islands located at the centre of the South Pacific region constitutes 7% of the region's population. However, approximately 40% of the region's total cumulative COVID-19 cases, as of 1 December 2021, were identified in Fiji [4]. The first case of the disease in Fiji was discovered and subsequently announced on 19 March 2020. The numbers went up slightly, but the spread of the virus was adequately controlled as a result of immediate travel bans, restricting public movement through targeted curfews, lockdowns, early investment in testing capacity and immediate closure of schools and non-essential businesses. No community transmissions were recorded after 18 April 2020. However, repatriation flights kept bringing in border cases that were kept in strict quarantine facilities before their release into the communities. It was from a breach in protocol at one of these quarantine facilities where on the on 19 April 2021, Fiji confirmed its first community case after 365 days of no community transmission of the virus. A more transmissible variant of the virus had been discovered in Fiji, the Delta variant which is up to 60% more transmissible than the previous variant [5]. The COVID-19 infection rate surged in Fiji and as of 1 December 2021 there have been 52 532 cases and 697 deaths in this second wave of the virus compared to 72 cases and 2 deaths from the first wave of the pandemic [6].

To mitigate the spread of the virus, targeted lockdowns were placed and movement restricted through curfews. The first batch of a 2-dose vaccine for arrived in Fiji on the 6th of March, 2021 and the first groups to receive their first dose of the vaccines were the frontline workers (airport workers, health front-liners, sea-ports, quarantine facility staff, hoteliers working in quarantine facilities, defence forces and some other essential workers), persons with existing medical conditions and the elderly. It was later administered to the general public and as of 1 December 2021, 601 400 people had already received their first dose and 560 570 were fully vaccinated. This corresponds to 97.3% and 90.7% of the target population (≥ 18 years old) receiving the first and second dose respectively [6].

In the global arena, as COVID-19 continuously crippled many economies and livelihoods, many researchers have published articles empathising epidemic forecasts that strongly relate to mathematical models. Epidemiologists have utilised these models to support a wide spectrum of policy questions globally [7]. Some recent work on modelling includes using the compartmental models such as SIR (Susceptible, Infected, Removed (recovered or deceased)) [3, 8, 9] or its extensions such as the SEIR (Susceptible, Exposed, Infectious, Recovered) [10, 11], SIRD (Susceptible, Infectious, Recovered, Dead) [12, 13] and the SEIRD (Susceptible, Exposed, Infected, Recovered, Dead) model [14, 15].

The present investigation utilises the Kermack–Mckendrick SIR model, one of the basic compartmental models, to evaluate and forecast the outbreak in Fiji. The compartment models assume that the population is homogenous, that is, each individual exhibits similar characteristics [16].

This study aims to:

- estimate the time-varying model parameters and to formulate a mathematical model to adequately understand the dynamics of the pandemic in Fiji,
- examine the impact of the control measures currently employed in Fiji, and
- use the model to validate and forecast the COVID-19 cases in Fiji.

The paper is structured as follows: In Section ‘Methods and materials’, the model and methodology are explained. In Section ‘Results’, we present the findings from the method used. Together with their validating reasons, these results are later described and conclusions are made in Section ‘Discussion’.

## Methods and materials

Several variations of the SIR model exist in the literature [3, 17–19]. Mungkasi [18] proposes a successive approximation method for solving the explicit SIR model with a constant vaccination strategy. Moreover, using an appropriate successive approximation method Mungkasi [17] obtains a superior explicit solution to the SIR model for dengue fever transmission. Cooper *et al*. [3] improved the classical SIR model with the ability to accommodate surges in the number of susceptible individuals in the population. The classical SIR model assumes a constant population where the susceptible population decreases monotonically towards zero. However, the authors in [3] proposed a model where the susceptible population is adjusted at various times to account for newly infected individuals.

Various studies [3, 8, 9, 17–21] have mainly investigated continuous or explicit time-discrete schemes. In contrast, Wacker and Schlüter [22] have analysed the properties of the implicit time-discrete SIR model, including nonnegativity and boundedness of solution, global existence and uniqueness in time, monotonicity properties and error analysis. We employed the implicit time-discrete SIR model [22] for short-time prediction and to keep modelling as interpretable as possible. The main reason for choosing the SIR model in the present study is its expediency and ease of implementation compared to other compartment models, along with high robustness in explaining the evolution of the pandemic.

**Fig. 2.**
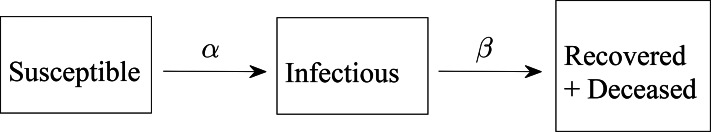
Illustration of the SIR model.

The SIR model is given by three coupled ordinary differential equations (ODEs) that describe the time evolution of our three main subpopulations [3]. The encounters between the individuals infected and susceptible occur at a rate proportional to their respective numbers in the population. The rate of new infections can thus be defined as *αSI*, where *α* is the effective transmission rate, *S*(*t*) is the number of people susceptible to the disease and *I*(*t*) represents the number of people infected, i.e. active cases. The number Infected (*I*) are assumed to recuperate with a constant probability at any time (*t*), which translates into a per capita recovery rate that we denote with *β*, and thus an overall rate of recovery *βI*. The transmission and recovery rate of any epidemic changes with vaccination and other damping measures such as lockdowns, compulsory mask usage in public and an increase in personal cleanliness [23]. Figure 2 shows the structure of the model based on the above assumptions.

The SIR model is the following system of ODEs [3, 22]:1
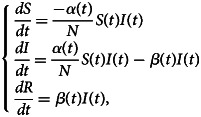
where *R*(*t*) is the number of people who have recovered or had deceased. *α*(*t*) and *β*(*t*) are the unknown time-varying model parameters. The total population under consideration is represented by *N*. The ODEs in Eq. (1) are interdependent as a closed population when a nation-wide disease outbreak is considered [3], and thus at any given time2
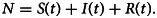


### Selection of model

The ODEs in the SIR model are discretised using the finite difference scheme. There are numerous works with mainly explicit schemes regarding time-discrete SIR models in literature [21, 24]; however Allen [20], and Wacker and Schluter [22] proposed an implicit time-discrete edition of this classical SIR model and showed that this time-discrete variant maintained various time-continuous properties. In this study, we follow the implicit numerical algorithm in [22] and discretise the SIR model as3
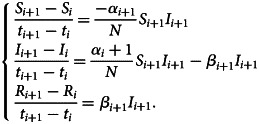


Assuming that our time interval [0, *T*] can be divided by a strictly increasing sequence 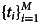 with *M* ∈ ℕ subintervals, Eq. 3 gives a fully implicit structure of the time-continuous SIR model for all *i* ∈ (1,  2,  … *M* − 1) and *N* = *S*_*i*+1_ + *I*_*i*+1_ + *R*_*i*+1_ = *S*_*i*_ + *I*_*i*_ + *R*_*i*_ [22].

### Selection and pre-treatment of COVID-19 data

**Fig. 3.**
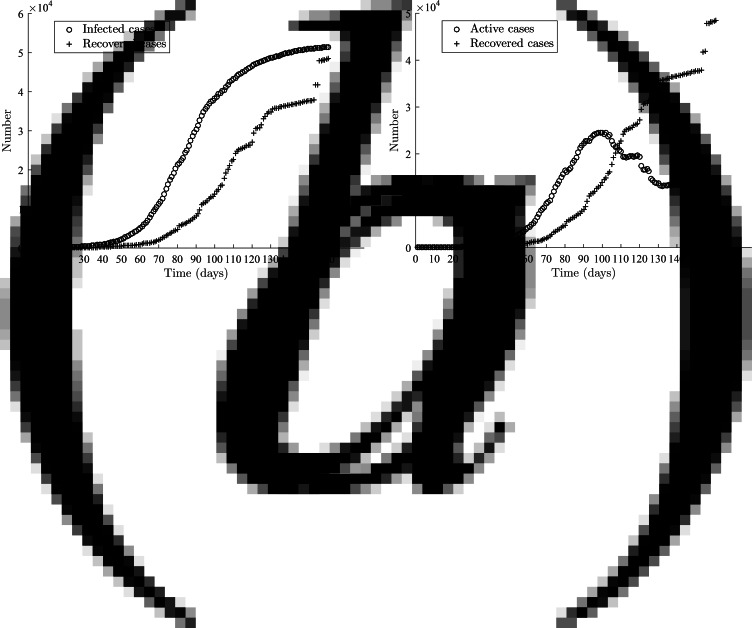
Unprocessed (observed) COVID-19 data for Fiji from *t*_1_ (4 May 2021) to *t*_160_ (10 October 2021). (a) shows the cumulative infection and cumulative recovered cases (*R*). (b) shows the daily active cases (*I*) and cumulative recovered cases (*R*).

The data for COVID-19 was acquired from the Fiji's Ministry of Health and Medical Services (MOHFiji) website (www.health.gov.fj) [6]. The data includes the cumulative number of infected cases (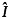), the cumulative number of recovered cases (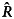) and the cumulative number of death cases (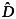) in Fiji. Following [22], we define 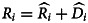 and 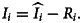 We considered the COVID-19 data of Fiji for the second wave of the pandemic from 4 May 2021 (*t* = 1) to 9 December 2021 (*t* = 220) of which the data corresponding to time 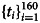 is used for estimating the parameters of the discrete-time SIR model and the data for time 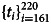 is used to validate the estimated model. Figure 3a shows the cumulative cases of reported infected people and the cumulative cases of reported recovered people in Fiji. Apparently, there are two jumps in the plot of total recoveries around days 152 and 154. The corresponding jumps are subsequently visible in Figure 3b showing the number of the active cases. Possible explanations for these sudden jumps would be late testing, identification and recording of recovered patient numbers.

**Fig. 4.**
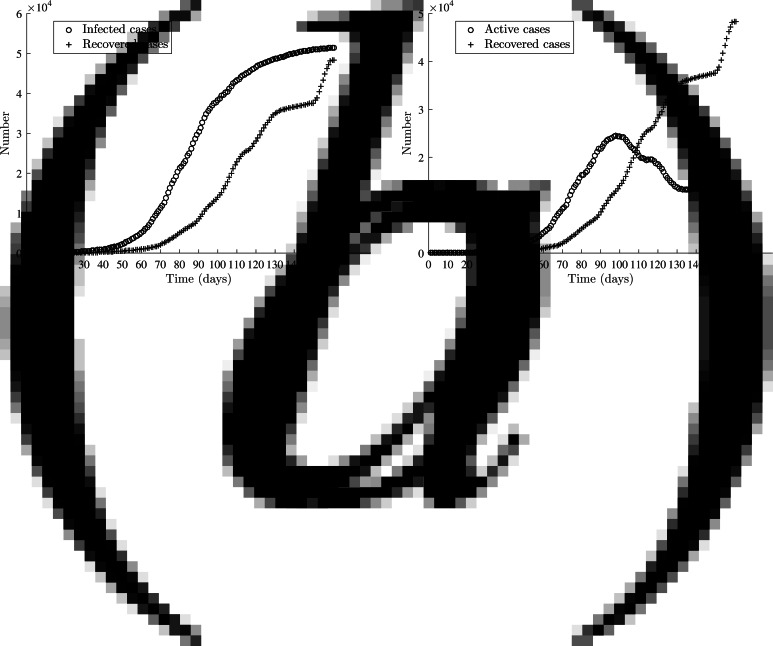
(a) Smoothening the recovery (*R*) curve with a 7MA filter, and (b) subsequently improving the curve for active cases (*I*).

A 3-day moving average (3MA) and a 5-day moving average (5MA) filter was employed by Law *et al*. [9] to smoothen daily infected data for Malaysia. Cartocci *et al*. [25] in their study on analysing gender and age-grouped data for Italy used the 7-day moving average (7MA) method to remove noise and excess variability from data. The authors in [9] and [25] also highlighted the importance of choosing a reasonable smoothing moving average window as a wider window would allow for a higher degree of noise filter, but meaningful rapid variations of the pandemic data would not be obtained. A 7-day window for the moving average filter is a fair compromise, and widely acceptable for smoothening noisy and erratic for the COVID-19 data as the mean incubation period of the virus is also 7 days [26]. For this study, a 7MA was applied to the recovered data, 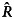, to eliminate sudden jumps and thus to obtain a smooth sequential data. The processed data using 7MA is shown in Figure 4, showing the improvement in smoothness and continuity in the recovered and hence the active cases.

### Calculation of time-varying *α* and *β* from observed data

We follow Wacker and Schlüter [22] and summarise the algorithm used to estimate the time-varying model parameters and the procedure adopted to forecast the COVID-19 cases in Fiji. The time-varying transmission and recovery rates are determined from Eq. (3) using discrete values of *I* and *R* observed at time *t*_*i*_ for *i* = 1,  …,  *M* − 1 where *M* = 160. Assuming *S*_*i*+1_ > 0 and *I*_*i*+1_ > 0, the time-varying parameters are computed as4
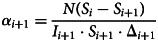
and5
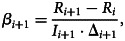
where Δ_*i*+1_ = *t*_*i*+1_ − *t*_*i*_ = 1 for *i* = 1,  …. *M* − 1.

After the time-varying model parameters are computed, the implicit time-discrete solution of the SIR model is estimated. Assuming that 0 < *α*_*i*_ < 1 and 0 < *β*_*i*_ < 1 are known for all *i* ∈ (1,  2,  … *M* − 1) and that the initial values of *S*_1_ > 0, *I*_1_ > 0 and *R*_1_ ≥ 0 are known, an implicit solution scheme of Eq. (3) reads6
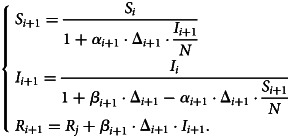


Assuming that *S*_*i*_ > 0, *I*_*i*_ > 0 and *R*_*i*_ ≥ 0, Eq. 6 is uniquely solvable for all *i* ∈ (1,  2,  … *M* − 1) [22]. Here, *I*_*i*+1_ for *i* = 1,  …,  *M* − 1 is first calculated using7
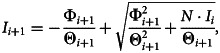
where Θ and Φ are defined as8
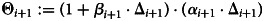
and9
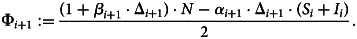


The solution scheme for *S*_*i*+1_ and *R*_*i*+1_ follows after computing *I*_*i*+1_. Note that *α*_*i*+1_ ≠ 0, which implies that Δ_*S*(*t*)_ = *S*_*i*+1_ − *S*_*i*_ ≠ 0 from (4).

The resulting implicit time-discrete scheme, i.e. Eq. 6, is the forward difference approximation of the SIR model, i.e. Eq. 3. The global existence, global uniqueness, non-negativity and boundedness of the solution, monotonicity properties, and error analysis of the implicit scheme have been well established. We refer the readers to [22] for a detailed analysis of the above properties. Additionally, the numerical solution implicit scheme is uniquely solvable for all time steps [22]. Moreover, the implicit time-discrete scheme is a rewritten version of the well-known implicit Eulerian time-stepping scheme, which is known to be unconditionally stable [27]. This method was implemented in [22] to successfully model the spread of COVID-19 in Germany and Iran.

### Model validation and short term forecast

**Fig. 5.**
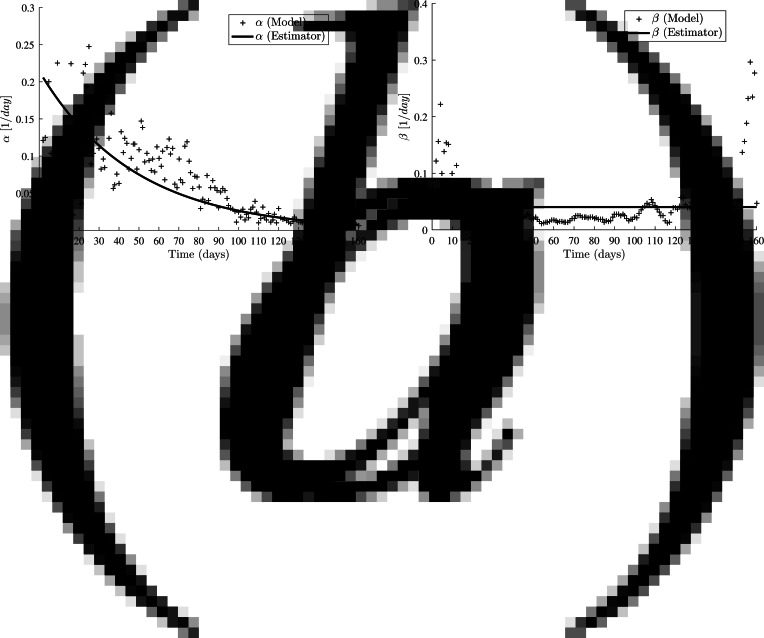
Time-varying transmission and recovery rates from processed data for Fiji. Superimposed are the estimator functions. (a) The estimated parameters are *α*_1_ ≈ 0.2144 and *α*_2_ ≈ 0.0210. (b) The estimated recovery rate is *β* ≈ 0.0403 (the mean value on the full interval).

To validate the model for 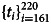 and make a short-term forecast of COVID-19 cases in Fiji, time-varying model parameters, for *t*_*i*_ (*i* > 220), are estimated following the approach in [22]. Through inspection of the time-varying transmission rate as shown in Figure 5a, it can be inferred that the model follows an exponential decay function. Similarly, except for a few areas of instability, as explained later in Section ‘Results’, the recovery rates are mostly constant for the duration of the study as seen in Figure 5b as well. Similar observations were made for COVID-19 cases in Germany and Iran [22], and Bulgaria [28]. Hence, the time-varying transmission and recovery rate is assumed to take the following form:10
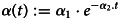
and11
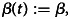
for *t* ≥ 1. The real constants *α*_1_, *α*_2_ and *β* which is are determined using the time-varying parameters 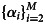
and 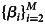 as defined by Eqs. (4) and (5).

Since both 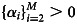 and 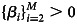 for *i* = 2,  … *M*, it is assumed that constants *α*_1_ > 0 and *β* > 0. The non linear relation in Eq. (10) is linearised using the parametric transformation12
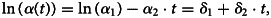
where *α*_1_: = ln(*δ*_1_) and *α*_2_: = −*δ*_2_ as in the case of maximum log-likelihood estimation (MLE). MLEs draw conclusions about the population most likely to have generated a sample, especially the joint probability distribution of the random variables {*y*_1_,  *y*_2_,  *y*_3_,  …,  *y*_*n*_}. This method of parameter estimation's many optimal properties and working algorithm have been thoroughly discussed in [29]. To find suitable estimators 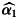, 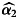 and 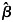 for *α*_1_, *α*_2_ and *β*, respectively, a cost function 
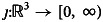
, is defined as:13
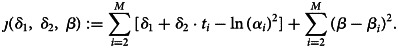


The solution rests in showing that the function 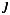 possesses a unique local minimiser 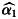, 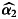 and 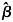. The local minimisers are obtained by setting all the partial derivatives of the cost function equal to zero. Hence, setting 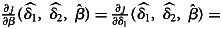 
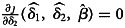
 yields [22]14
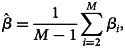
15
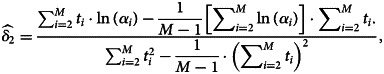
and16
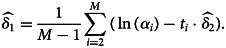


Using the local minimisers (Eqs. (14)–(16)) and the estimated time-varying model parameters (Eqs. (10) and (11)), the estimated model is validated for 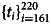. In the absence of an absolute standard method in forecast verification, the model's performance has been done in line with Lal *et al*. [13] and Salgotra *et al*. [30]. The model's accuracy was evaluated by computing the Relative Mean Absolute Error (RMAE) of the simulated model state as17
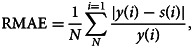
where *y*(*i*) is the observed state, *s*(*i*) is the simulated state using the modelled parameters, and *N* is the size of the observed data. Willmott and Matsuura [31] indicate that RMAE is a more natural and accurate measure of average error, and (unlike RMSE or related measures) is unambiguous. It can be further stated that for a model to be reliable and accurate, the correlation coefficient between the desired and projected values must be strong [32]. Hence the coefficient of determination, *r*^2^, values are calculated, using [13, 32]18
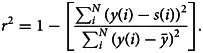


Furthermore, short term (30 days) forecast is made for the cumulative number of infected and recovered COVID-19 cases in Fiji. In this study, all computations and simulations were performed using MATLAB R2016a software.

### Time-varying basic reproduction number

The basic reproduction number 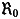 of an infectious disease is the number of people who contract the disease from an infected person assuming the whole population is susceptible [20, 33]. In the SIR model, the time-varying basic reproduction number is computed as19
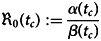
for arbitrary *c* ∈ (1,  2,  … *M*) and assuming *β*(*t*_*c*_) > 0 [33].

## Results

The time-varying COVID-19 transmission (*α*) and recovery rate (*β*) for Fiji is presented in Figure 5. Clearly, the transmission rate decreased due to corrective measures such as localised lockdowns, initial bans and restrictions on social gatherings, compulsory cladding of proper masks in public, and a gradual increase in vaccination rates. However, the transmission rates are volatile at the beginning but gradually stabilise after *t*_80_. A significant cause for this instability could be attributed to untimely COVID testing and analysis. Test samples are sent from health facilities around the country to Fiji Centre for Disease Control (Fiji CDC) in the capital city for testing, and results are obtained within 48 h after the swab tests. This non-decentralising of the COVID testing results in delays, backlogs and placement of the positive results in incorrect time bins. In late May 2021, 11 000 swab samples were sent to Australia as testing facilities in Fiji were inundated with testing samples and were severely backlogged [6]. Moreover, there are unknown cases of transmission as people with a mild course of the disease or asymptomatic infections may not have come forward for testing.

Recovery rates being stagnant for certain days and abruptly rising on certain days in the beginning of the pandemic in Fiji suggests batch testing for recovered patients. This may explain the volatility in the recovery rates at the start of the second wave of the virus. The rate seems to be constant, thereafter with moderate deviations due to increases in test capacity and regularity.

**Fig. 6.**
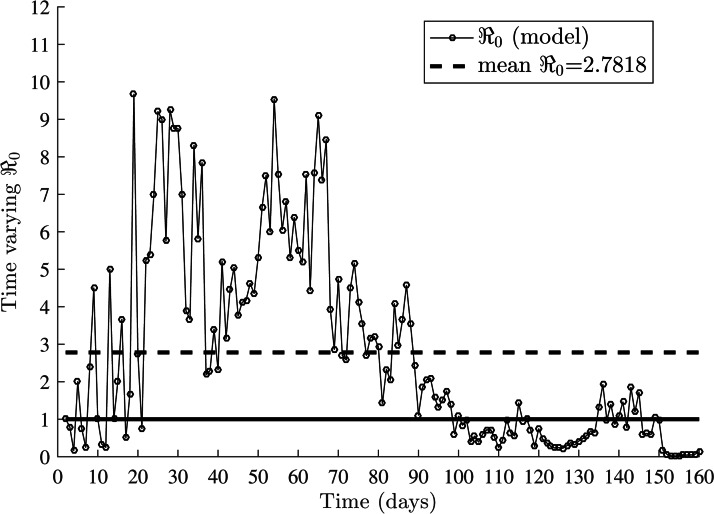
Time-varying, and average effective reproduction number from processed data for Fiji from *t*_1_ = 1 (4 May 2021) to *t*_*M*_ = 160 (10 October 2021).

The time-dependent reproduction number 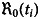 is shown in Figure 6. The computations produce high numerical 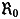
s in the beginning as there were only a few recovered cases at the start of the disease outbreak. The reproduction number further increased from early June 2021 to mid-July 2021. This increase can be attributed to rapid numbers of active cases as a result of the discovery and formation of COVID-19 clusters in many informal settlements in Suva and Nadi, such as Kinoya, Navosai, Nawaka, Tramline-Nadi, Waila and Grantham Road [6]. Although these areas were cordoned off when transmissions were discovered, the close proximity of dwellings in this area resulted in surges of infected cases within the area. Transmissions were also discovered in a few highly populous government institutions such as the navy headquarters, the Nasinu police barracks and the Suva's Colonial War Memorial Hospital [6]. The number of active cases peaked in mid-August and regressed thereafter, translating to monotonically lower values of 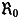.

**Fig. 7.**
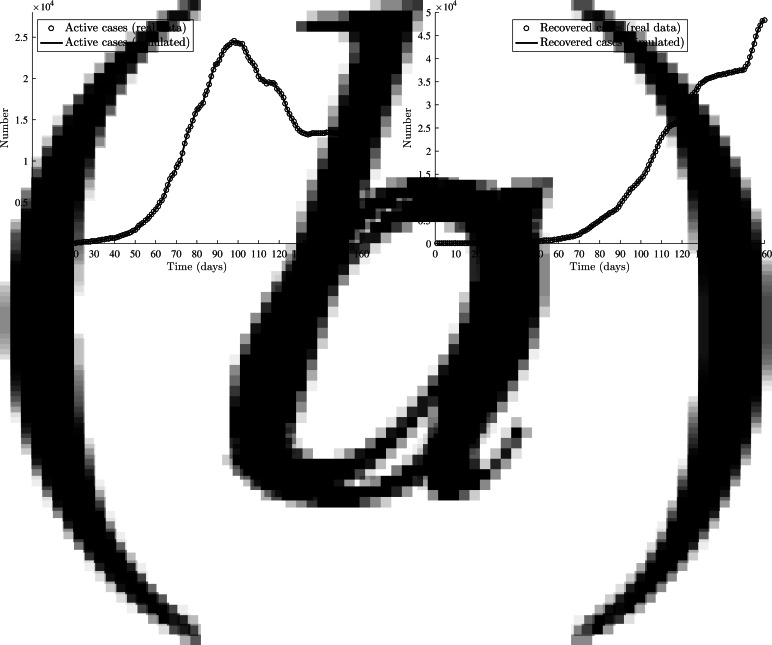
The 7MA processed data and implicit time-discrete SIR solution scheme for 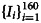 shown in (a) and 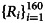 in (b).

Using the transmission and recovery rates from Eqs. (4) and (5), the time-discrete implicit SIR solution scheme in Eq. (6) is applied to the treated data for *I* and *R* for the considered period. The simulated active and recovered cases are illustrated in Figure 7.

**Fig. 8.**
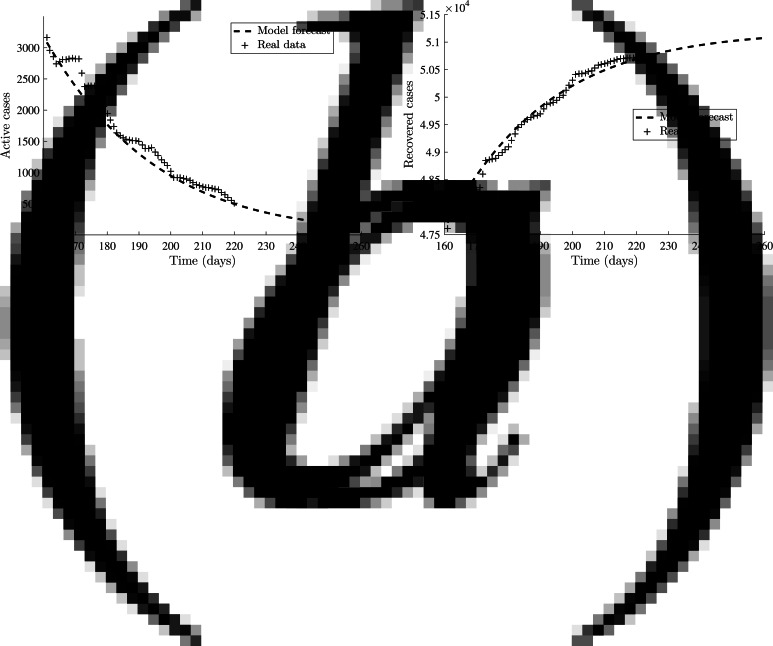
(a) Using the 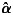 and 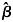 functions together with initial conditions *S*_160_ and *I*_160_ to validate the active cases, 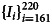. (b) The estimator functions and initial conditions *R*_160_, *I*_161_ are used to validate the recovered cases, 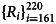. The model forecast is shown for the next 30 days i.e. 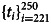.

The model forecast for the active cases and total recovered cases for 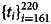 is compared against observed values in Figure 8. We observe a good fit between the anticipated and actual data. Table 1 shows the model-forecast active and recovered case numbers for the next 30 days i.e. from *t*_221_ = 10 December 2021 to *t*_250_ = 8 January 2022. Table 2 lists the RMAE and *r*^2^ values of the model states simulated using the estimated parameters. The performance of a proposed model in any scientific domain is exceptional if the RMAE is less than 0.05, good if the RMAE is between 0.05 and 0.1, and reasonable if the RMAE is between 0.1 and 0.2 [34]. The model forecast for the active cases, *I* and the recovered cases, *R* is good. This is also highlighted in the respective values of *r*^2^. The RMAE and the *r*^2^ values validate the quality of the proposed models and hence increase the chances of reliable predictions.

**Table 1.** Forecast for COVID-19 active (*I*) and recovered cases (*R*) in Fiji from *t*_221_ = 10 December 2021 to *t*_250_ = 8 January 2022

Date	*i*	*I*_*simulated*_	*R*_*simulated*_
10/12/2021	221	481	50 710
11/12/2021	222	465	50 726
12/12/2021	223	450	50 742
13/12/2021	224	436	50 758
14/12/2021	225	422	50 772
15/12/2021	226	408	50 787
16/12/2021	227	395	50 801
17/12/2021	228	382	50 814
18/12/2021	229	369	50 827
19/12/2021	230	357	50 840
20/12/2021	231	346	50 852
21/12/2021	232	334	50 864
22/12/2021	233	324	50 875
23/12/2021	234	313	50 886
24/12/2021	235	303	50 897
25/12/2021	236	293	50 907
26/12/2021	237	283	50 917
27/12/2021	238	274	50 927
28/12/2021	239	265	50 936
29/12/2021	240	256	50 945
30/12/2021	241	248	50 954
31/12/2021	242	240	50 963
01/01/2022	243	232	50 971
02/01/2022	244	224	50 979
03/01/2022	245	217	50 986
04/01/2022	246	210	50 994
05/01/2022	247	203	51 001
06/01/2022	248	196	51 008
07/01/2022	249	189	51 014
08/01/2022	250	183	51 021

**Table 2.** Model validation

	Active cases (*I*)	Recovered cases (*R*)
RMAE	0.1026	0.0019
*r*^2^	0.9626	0.9875

RMAE and *r*^2^ values of the simulated states.

## Discussion

A time-discrete SIR model is utilised for the modelling and forecasting the spread of the COVID-19 epidemic in Fiji by considering publicly available data from 4 May 2021 to 9 December 2021. The choice of the data selection dates corresponds to the second wave of COVID-19 cases in Fiji. We assumed an exponential decay model for transmission rates as observed in our experimental findings for time-varying *α* rates. However, the model for recovery rates was less evident from the calculated time-varying *β* values. The calculated recovery rates are volatile in Figure 5b at the beginning and towards the end (the reasons for which is mentioned in Section ‘Results’), the rates were primarily constant.

In this study, we have used a constant recovery rate, but in reality, recovery rates vary. Factors such as changes in socioeconomic factors, spending levels in medical infrastructure, the proportion of infected elderlies have effects on rates of recoveries [35]. As patients of different degrees of symptoms present themselves to medical authorities, the recovery rates of these cases will vary as well. Other studies, e.g. Lal *et al*. [13] and Hong and Li [36] used time-varying recovery rates. This is an obvious limitation of our study. A more accurate and reflective recovery rate model would in turn, produce far more improved results.

These estimated functions for the transmission and recovery rates were used to validate the active and recovered cases for October to mid-December 2021 and the forecast done for the next 30 days. The statistical results show that the proposed time-discrete SIR model is reliable, and new predictions can be derived based on this model.

The time-varying basic reproduction number, 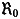 serves as the best proxy for disease progression [37]. Computing the reproduction number regularly and frequently is vital to comprehend the epidemic's trajectory and make real-time assessments of its scale. Furthermore, it is a critical criterion for evaluating the efficacy of current public health measures and planning future actions as needed [37, 38]. A value of 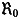 above 1 implies exponential growth in the number of cases of the disease in the population, the higher the value of 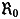 the harder it is to stop the outbreak. A value of 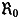 below 1 means that the outbreak is under control and will eventually stop [38]. The variations in 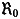 for Fiji is similar to the trend in transmission rates. The values are generally below one after 11 August 2021.

**Fig. 9.**
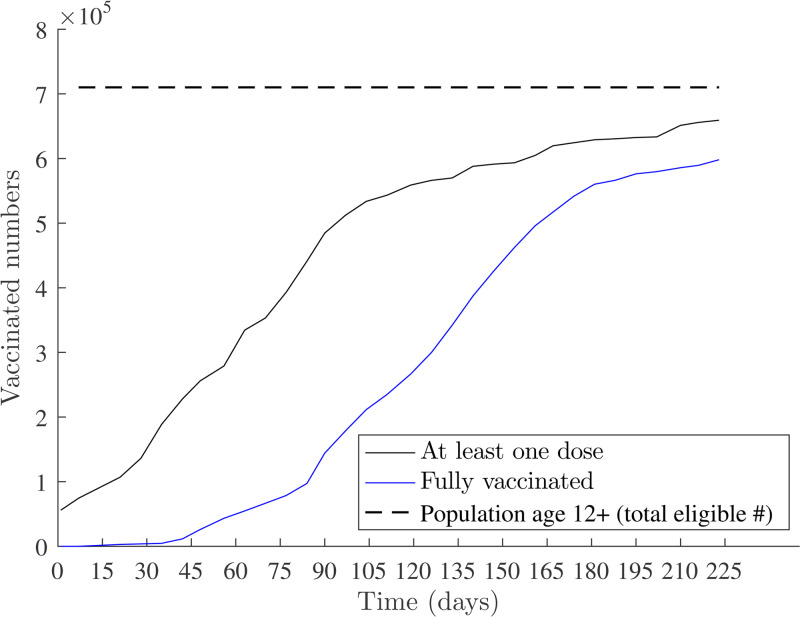
Numbers vaccinated in Fiji from *t*_1_ = 4 May 2021 to *t*_220_ = 9 December 2021.

Behavioural changes (e.g. regular hand-washing, wearing of masks, social distancing) and control interventions (e.g. school closures, market closures) seem to dampen disease transmission rates. Still, the most significant attribution to reduced disease transmissions is by increasing herd immunity through timely vaccinations [39]. Figure 9 illustrates the COVID-19 vaccination levels in Fiji [6]. As of 10 December 2021, 565 181 adults (of age ≥ 18) had been fully vaccinated with two doses. This corresponds to 91.4% of the adult population. The rollout of vaccinations for children aged 15–17 years started on 16 September 2021 and later made accessible for children aged 12–14 years on 15 November 2021 [6]. Booster doses of the vaccine for the target population who had been fully vaccinated for five months and above was administered from 29 November 2021 [6]. Vaccination rates for Fiji are high relative to neighbouring PICTs. Papua New Guinea had 5%, French Polynesia had 67% and New Caledonia had 75% vaccine coverage in the same period [4].

At this point, we note that the SIR model did not take into account many factors that play an essential role in the dynamics of viral diseases, such as the effect of the incubation period in the transmission dynamics, the impact of the measures already taken to combat the epidemic and the characteristics of the population (e.g. the effect of the age, gender, existing health conditions of people). Also, a subset of actual infected cases, which recorded mild or no symptoms of the virus, may not have presented themselves in local health centres for proper diagnosis, and thus their numbers do not appear in governmental statistics. This under-reporting of COVID-19 cases is much more prevalent during the localised influenza seasons as mild symptoms of COVID-19 and flu are generally similar [40]. This under-discovery and under-reporting of the total active COVID-19 cases inevitably distort epidemiologic reality, primarily for nations with low population values like Fiji. Also, compartmental models in epidemiology assume a homogeneous and closed population set. This will change when international borders are opened, and there is an influx of visitors. Fiji is a popular tourist destination for global travellers, with yearly visitor arrivals exceeding the nations local population. This will significantly change the model's dynamics. The presence of future variants of the virus may also substantially change rates of transmission and recovery.

Given the immense impact COVID-19 has had on small developing nations like Fiji, the relevant authorities must take the necessary decisive and collective action to mitigate risks and exposure to the virus. The outputs of the model employed in this study can help determine the need, or success of existing COVID countermeasures employed. The progress of infection spread can also be predicted based on real-time infectious disease data. The results from this study can be used by researchers, the Fijian government, and the relevant health policy makers in making informed decisions should a third COVID-19 wave occur.

Our research cannot predict when another COVID-19 outbreak will occur. However, when a new wave emerges, the model may be used to forecast the outbreak's size and severity. This is based on the model's ability to fit existing data. The progress of infection spread can also be predicted based on real-time infectious disease data. Finally, since the model overlooks the exact impact of vaccination on disease transmission in Fiji, further study is warranted to investigate how the vaccine deployment affects transmission rates and the appearance of future waves.

## Data Availability

The data that support the findings of this study is publicly available on the Ministry of Health and Medical Services, Fiji website (https://www.health.gov.fj/). Clarifications can be made on request from the corresponding author (RAS).

## Supplementary material

For supplementary material accompanying this paper visit https://doi.org/10.1017/S0950268822000590. click here to view supplementary material
